# Role of Artificial Intelligence in the Diagnosis of Oral Squamous Cell Carcinoma: A Systematic Review

**DOI:** 10.7759/cureus.81800

**Published:** 2025-04-06

**Authors:** Tanay Chowdhury, Pratik Kasralikar, Abdul Aleem Syed, Ramakrishna Tumati, Sandipkumar Patel, Dheeraj Kommineni

**Affiliations:** 1 Data Science, Amazon Web Services Generative AI Innovation Center, Sammamish, USA; 2 Business Administration, Lindsey Wilson College, Columbia, USA; 3 Technical Product Management, FHN Financial, Texas, USA; 4 Software and Advanced Technology Group (SATG), Intel, Beaverton, USA; 5 Computer Engineering, Gujarat Technological University, Ahmedabad, IND; 6 System Analytics, Hanker System, Chantilly, USA

**Keywords:** artificial intelligence, cancer diagnosis, machine learning, oral cancer, oral squamous cell carcinoma

## Abstract

Oral squamous cell carcinoma (OSCC) is a serious worldwide health issue. Early OSCC identification by the analysis of digital oral photos is possible with the combination of artificial intelligence (AI) and computer vision. The purpose of this systematic review was to evaluate the current evidence on the role of AI in the diagnosis of OSCC, focusing on the diagnostic performance, methodologies employed, and potential limitations of AI applications in this context. We followed the Preferred Reporting Items for Systematic Reviews and Meta-Analyses (PRISMA) guidelines to search for relevant studies across PubMed, Scopus, Web of Science, and Cumulative Index to Nursing and Allied Health Literature (CINAHL). In these databases, we found 286 studies, which were first screened for duplicates and then assessed on inclusion and exclusion criteria. Only 11 studies were found most relevant and were included in this study. These studies were also assessed for risk of bias using the Quality Assessment of Diagnostic Accuracy Studies 2 (QUADAS-2) tool. Numerous studies have shown impressive results for this job, frequently covering about 1000 photos and regularly reaching sensitivity rates above 85% with accuracy rates above 90%. The review examines these research in detail, providing insight into their methods, which include the application of contemporary machine learning and pattern recognition techniques in conjunction with various supervision techniques. However, because various datasets are utilized in different articles, it can be difficult to compare the results. In light of these results, this study emphasizes how urgently the area of OSCC detection needs more solid and trustworthy datasets. Additionally, it emphasizes how sophisticated methods like ensemble learning, multi-task learning, and attention mechanisms can be used as essential instruments to improve the sensitivity and accuracy of OSCC identification in oral photos. Together, these observations highlight how AI-driven methods for early OSCC diagnosis have the potential to greatly enhance patient outcomes and medical procedures.

## Introduction and background

Oral squamous cell carcinoma (OSCC) represents a significant global health challenge, being the most prevalent malignancy of the oral cavity [1]. In 2022, there were approximately 389,846 new cases of mouth and oral cancer worldwide, making it the 16th most common cancer globally [2]. Notably, countries such as India, China, and the United States reported the highest number of cases, with India alone accounting for 143,759 new cases [3].

Early detection of OSCC is crucial for improving patient outcomes, as delayed diagnosis often leads to advanced disease stages with poorer prognoses [4]. Traditional diagnostic methods, including visual examinations and biopsies, are subject to limitations such as interobserver variability and potential delays in diagnosis. In recent years, artificial intelligence (AI) has emerged as a promising tool in medical diagnostics, offering the potential to enhance accuracy and efficiency in disease detection [5].

Recent advancements in AI-driven diagnostic tools have significantly improved OSCC detection. Machine learning models, particularly deep learning architectures such as convolutional neural networks (CNNs), have demonstrated high accuracy in analyzing histopathological and imaging data [3]. Additionally, AI-powered oral cytology screening and computer-aided diagnostic (CAD) systems have been developed to automate lesion classification, reducing reliance on human expertise and minimizing diagnostic delays. The integration of AI with digital pathology, radiomics, and multimodal data fusion is further enhancing precision in OSCC detection [5].

Several studies have explored the application of AI in the detection of OSCC. A systematic review and meta-analysis encompassing 16 studies with a total of 6,606 samples evaluated the accuracy of AI-assisted technologies in detecting OSCC [6]. The findings revealed a summary sensitivity of 92% and a summary specificity of 91.9%, indicating that AI systems can detect OSCC with high accuracy [6].

Recent developments also highlight the potential of AI-driven biomarkers and predictive analytics in OSCC prognosis. AI models leveraging genomic and transcriptomic data are being explored for risk stratification and personalized treatment planning [4]. Furthermore, real-time AI-assisted intraoperative assessment is gaining traction, aiding in surgical margin evaluation and improving patient management [2].

Despite these promising results, the integration of AI into clinical practice for OSCC diagnosis requires further validation through large-scale, prospective studies [7]. Addressing challenges such as data standardization, model interpretability, and integration into existing clinical workflows is essential for the successful adoption of AI technologies in this field.

This systematic review aims to comprehensively evaluate the current evidence on the role of AI in the diagnosis of OSCC, focusing on the diagnostic performance, methodologies employed, and potential limitations of AI applications in this context.

## Review

Methodology

Protocol

This systematic review was conducted following the guidelines outlined in the Preferred Reporting Items for Systematic Reviews and Meta-Analyses (PRISMA) statement [8].

Eligibility Criteria

The inclusion and exclusion criteria were established to ensure the selection of relevant studies investigating the role of AI in diagnosing OSCC. The criteria are summarized in Table 1.

**Table 1:** Inclusion and exclusion criteria OSCC: oral squamous cell carcinoma; AI: artificial intelligence

Criteria	Inclusion	Exclusion
Population	Studies involving human subjects diagnosed with OSCC	Studies involving animals or in vitro models
Intervention	Studies employing AI models for OSCC diagnosis	Studies not involving AI-based diagnostic tools
Comparator	Control groups with benign, malignant, or normal mucosa conditions	Studies without a comparator group
Outcome measures	Diagnostic performance metrics such as accuracy, sensitivity, and specificity	Studies lacking performance evaluation of AI models
Study design	Original research articles (prospective or retrospective studies, clinical trials)	Reviews, editorials, case reports, conference abstracts
Language	English	Non-English studies without available translations

Information Sources

A comprehensive literature search was conducted in four electronic databases: PubMed, Scopus, Web of Science, and Cumulative Index to Nursing and Allied Health Literature (CINAHL). The search was performed from database inception to March 18, 2025, to identify relevant studies evaluating AI applications in OSCC diagnosis.

Search Strategy

A combination of Medical Subject Headings (MeSH) terms and free-text keywords were used to maximize search sensitivity. Boolean operators (AND, OR) were applied to refine the search strategy. The search strategy for each database is detailed in Table 2.

**Table 2:** Search strings for each database CINAHL: Cumulative Index to Nursing and Allied Health Literature

Database	Search string
PubMed	("Artificial Intelligence" OR "Machine Learning" OR "Deep Learning") AND ("Oral Squamous Cell Carcinoma" OR "OSCC") AND ("Diagnosis" OR "Detection")
Scopus	("Artificial Intelligence" OR "AI" OR "Neural Networks") AND ("Oral Cancer" OR "Squamous Cell Carcinoma") AND ("Screening" OR "Diagnosis")
Web of Science	("Machine Learning" OR "Deep Learning" OR "AI-based Diagnosis") AND ("Oral Neoplasms" OR "OSCC") AND ("Histopathological Analysis" OR "Image Processing")
CINAHL	("AI in Cancer Diagnosis" OR "Computer-Aided Diagnosis") AND ("Oral Malignancy" OR "OSCC") AND ("Sensitivity and Specificity")

Study Selection

All retrieved records were imported into Endnote X9 (Clarivate, London, United Kingdom), a reference management software, and duplicates were removed. Two independent reviewers screened the titles and abstracts for relevance. Full-text articles of potentially eligible studies were retrieved and assessed for final inclusion based on the predefined eligibility criteria. Discrepancies were resolved through discussion or consultation with a third reviewer.

Data Extraction

A standardized data extraction form, in an Excel sheet (Microsoft Corporation, Redmond, Washington, United States), was developed to collect key study characteristics, including author, publication year, sample size, AI model used, comparator groups, and diagnostic performance metrics (accuracy, sensitivity, specificity). Data extraction was conducted independently by two reviewers to ensure accuracy and consistency.

Risk of Bias Assessment

The risk of bias in included studies was evaluated using an appropriate tool, such as the Quality Assessment of Diagnostic Accuracy Studies 2 (QUADAS-2) tool. The assessment considered four domains: patient selection, index test, reference standard, and flow and timing. Studies were categorized as having a low, moderate, or high risk of bias.

Results

Search Results

**Figure 1:**
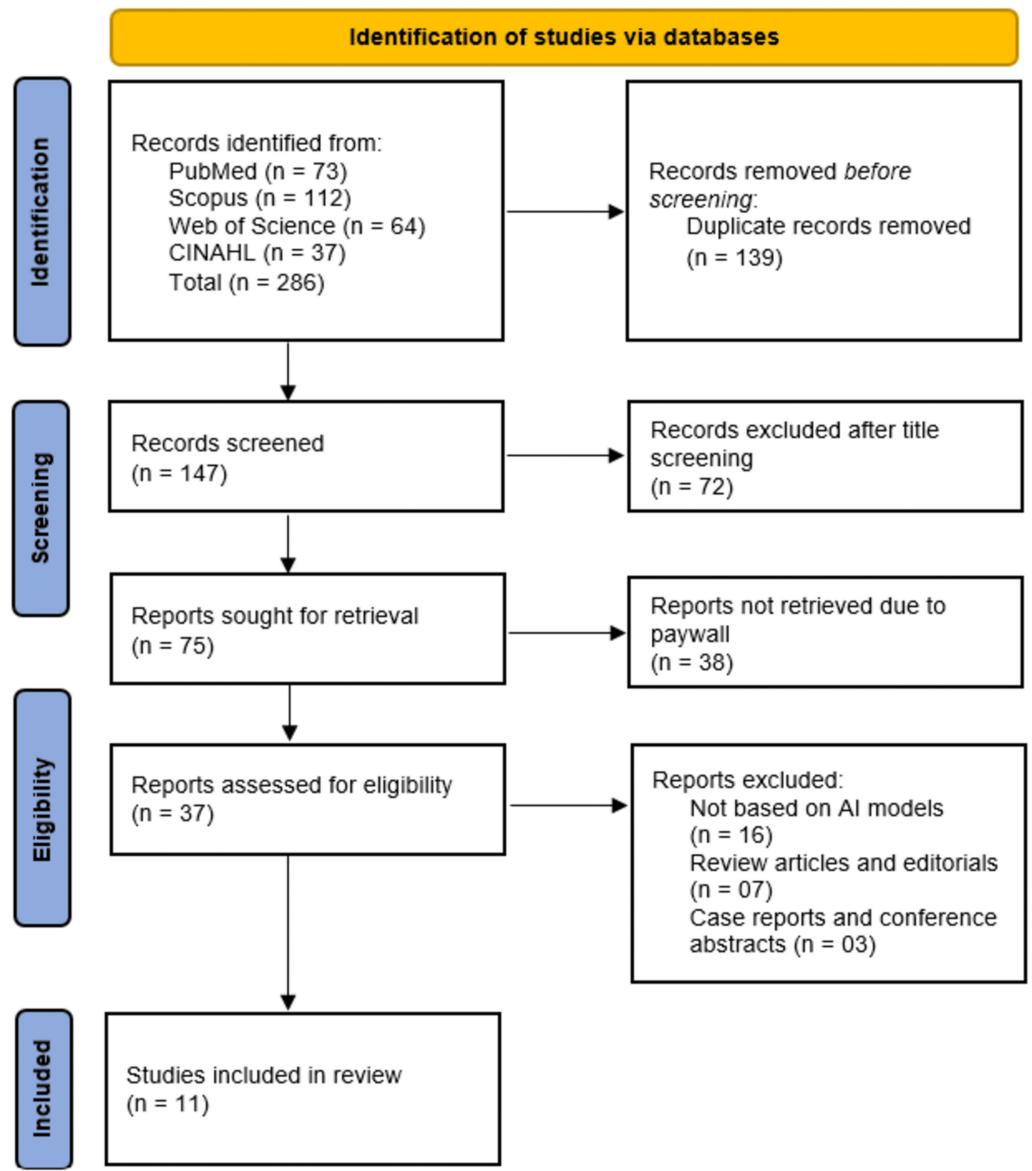
PRISMA flowchart of study selection PRISMA: Preferred Reporting Items for Systematic Reviews and Meta-Analyses; CINAHL: Cumulative Index to Nursing and Allied Health Literature; AI: artificial intelligence

A total of 286 records were identified through database searches, including 73 from PubMed, 112 from Scopus, 64 from Web of Science, and 37 from CINAHL. After removing 139 duplicate records, 147 studies proceeded to title and abstract screening. Of these, 72 studies were excluded based on relevance, leaving 75 studies for full-text retrieval. However, 38 studies could not be retrieved due to paywall restrictions, reducing the number of studies assessed for eligibility to 37. Following a detailed evaluation, 16 studies were excluded for not being based on AI models, seven studies were excluded as review articles or editorials, and three studies were excluded as case reports or conference abstracts. Ultimately, 11 studies met the inclusion criteria and were included in this systematic review (Figure 1).

Characteristics of the Included Studies

This systematic review included 11 studies that evaluated the application of AI in diagnosing OSCC. The sample sizes in the included studies varied considerably, ranging from 35 patients [9] to 7,148 patients [10]. Most studies used a combination of benign and malignant cases as control groups, while some, such as Welikala et al. [11,12] and Tanriver et al. [13], specifically included oral potentially malignant disorders (OPMDs) as part of their study cohorts [14,15].

The studies employed different AI models for diagnostic assessment, with CNNs being the most commonly used architecture. Among the deep learning frameworks, ResNet, VGG19, NASNet, EfficientNet, and MLSO+SVM were frequently utilized. Welikala et al. [11] incorporated clinically guided attention mechanisms, while Huang et al. [16] optimized CNN with an improved salp swarm algorithm (ISSA). Jurczyszyn et al. [9] used a probabilistic neural network, which differs from conventional CNN-based architectures.

The diagnostic performance of AI models was assessed through accuracy, sensitivity, and specificity. Different AI architectures, including CNNs, support vector machines (SVMs), and deep learning-based ensemble models, were utilized for OSCC detection, each demonstrating varying levels of diagnostic precision. The reported accuracy ranged from 80.88% [12] to 98% [10,17], highlighting the strong capability of AI models in identifying OSCC. Sensitivity values varied between 85.71% [12] and 100% [9,10], indicating the effectiveness of AI in detecting malignant cases. Specificity values ranged from 76.42% [11] to 99.6% [10], suggesting some variability in AI models' ability to correctly identify non-malignant cases.

CNN-based models were particularly effective in histopathological image analysis, leveraging deep feature extraction to improve classification accuracy. Meanwhile, SVMs and hybrid AI approaches integrated clinical and imaging data to enhance diagnostic reliability. Despite these advancements, challenges remain, including variations in training datasets, differences in preprocessing techniques, and the need for external validation to ensure model generalizability across diverse populations. However, some studies, including Huang et al. [16], Alanazi et al. [18], and Tanriver et al. [13], did not report specificity, which limits comparative analysis (Table 3).

**Table 3:** Characteristics of the included studies and performance matrix of different AI models AI: artificial intelligence; CNN: convolutional neural network; ISSA: improved sparrow search algorithm; MLSO: multi-objective Lichtenberg search optimization; SVM: support vector machine; NASNet: neural architecture search network; OPMD: oral potentially malignant disorder; VGG: Visual Geometry Group; ResNet: residual neural network; VGG-Skip: VGG with skip connections; EfficientNetB4: efficient neural network model (B4 variant)

Author	Publishing year	Sample size	Controls used	AI model	Performance		
					Accuracy (%)	Sensitivity (%)	Specificity (%)
Fu et al. [15]	2020	1469	Benign and malignant	CNN	92.3	90	93.5
Jurczyszyn et al. [9]	2020	35	Malignant and normal mucosa	Probabilistic neural network	Not reported	100	97
Huang et al. [16]	2023	105	Benign and malignant	CNN+ISSA	97.33	87.34	Not reported
Ding et al. [14]	2023	105	Benign and malignant	MLSO+SVM	96.94	97.7	92.37
Alanazi et al. [18]	2022	105	Benign and malignant	NASNet	92.59	95	Not reported
Welikala et al. [12]	2020	1180	Benign and malignant OPMD	VGG19	80.88	85.71	76.42
Welikala et al. [11]	2021	2155	Benign and malignant	VGG with clinically guided attention	83.3	Not reported	Not reported
Xue et al. [10]	2022	7148	Benign and malignant	ResNet	98	100	99.6
Shamim et al. [17]	2022	200	Benign and malignant	VGG19	98	89	97
Tanriver et al. [13]	2021	162	Benign and malignant OPMD	EfficientNetB4	87	86	Not reported
Nanditha et al. [19]	2021	332	Benign and precancerous	ResNet50 VGG-Skip	96.2	98.14	94.23

Risk of Bias Assessment Results

The risk of bias assessment identified variations in study quality across the included studies. Jurczyszyn et al. [9] had a high risk of bias in patient selection due to a small sample size (n=35), which may limit the generalizability of findings. Welikala et al. [11] and Tanriver et al. [13] had unclear risk in the index test domain, as the AI models used were not externally validated with independent datasets. Huang et al. [16] and Ding et al. [14] showed low bias in the reference standard domain, as they used histopathological confirmation for diagnosis. However, Alanazi et al. [18] and Shamim et al. [17] presented concerns in the flow and timing domain, as missing data handling was not explicitly reported. Overall, while most studies demonstrated low bias in reference standards, inconsistencies in sample size, external validation, and data handling suggest the need for more rigorous study designs to improve the reliability of AI-based OSCC diagnostics (Table 4).

**Table 4:** Risk of bias assessment using the QUADAS-2 tool QUADAS-2: Quality Assessment of Diagnostic Accuracy Studies 2

Study	Patient selection	Index test	Reference standard	Flow and timing	Overall bias
Fu et al. [15]	Low	Low	Low	Low	Low
Jurczyszyn et al. [9]	High	High	High	High	High
Huang et al. [16]	Low	Low	High	Low	Moderate
Ding et al. [14]	Low	Low	Low	Low	Low
Alanazi et al. [18]	Low	Moderate	High	Low	Moderate
Welikala et al. [12]	Low	Moderate	High	Low	Moderate
Welikala et al. [11]	Low	Moderate	High	Low	Moderate
Xue et al. [10]	Low	Low	Low	Low	Low
Shamim et al. [17]	Low	Low	Low	Low	Low
Tanriver et al. [13]	Low	Moderate	High	Low	Moderate
Nanditha et al. [19]	Low	Low	Low	Low	Low

Discussion

Despite a lack of accessible data, the use of AI in OSSC detection has produced encouraging results, with F1 scores of 94.9% and accuracy levels of up to 99% [10,13,15-18]. These accomplishments are especially noteworthy in light of the very small dataset sizes, an average of 1200 photos per study, with the largest dataset having 7148 images and the lowest having only 105. It is imperative to recognize that gathering data for medical imaging is intrinsically more time-consuming and resource-intensive, especially when considering OSCC.

A number of crucial tactics have been developed to improve oral cancer detection performance [20,21]. Even though attention mechanisms are still being investigated, their introduction shows promise as a workable strategy. By concentrating computing resources on pertinent visual regions, a recent study that included attention mechanisms demonstrated the new possibilities that emerge, greatly enhancing detection skills [14]. Furthermore, recent studies demonstrate that attention transformers and similar models, which typically require large amounts of data, can be effectively trained on relatively small datasets. Additionally, ensemble learning has shown a lot of promise. A noteworthy example demonstrated the synergistic advantages of combining multiple models for improved categorization, achieving an astounding accuracy rate of 96.2% [12]. Even with fewer datasets, this method makes use of the variety of separate models to produce predictions that are strong when combined [13]. Techniques for improving images have become a notable way to increase detection accuracy. These methods have been successful in improving image quality, which has resulted in better model performance, even though they have been applied to comparatively smaller datasets [9,11].

An innovative method that makes use of anatomical cues to direct the detection process is the integration of feature detection [22]. Using multi-task learning improves cancer detection accuracy, perhaps leading to more precise and focused diagnoses [11,19]. Out of all the architecture options, VGG-19 performs very well, continuously producing the top outcomes in numerous studies. The general high accuracy rates seen are a result of its capacity to extract intricate features from images, which is in line with the requirements of oral cancer detection [16,18].

However, there aren't many datasets available for this use. Additionally, there is no biopsy diagnosis verification in the reference benchmark dataset for oral cancer detection, which is rather small. Consequently, preprocessing and data augmentation are crucial [10]. An interesting strategy is to use methods like adaptive histogram equalization and image enhancement using discrete wavelet transform. There is obviously a lot of space for development and additional study, even using common data augmentation methods like rotation, flipping, and cropping [10,12,14].

One significant limitation that is commonly noted is the lack of uniformity in the datasets used, which makes it more difficult to compare various studies. The most widely used benchmark dataset, which comes from Kaggle, is rather tiny and has shown reliability problems [23]. The results of research employing this dataset might, therefore, not be easily generalizable to other contexts.

Although using AI to detect oral cancer has potential, it is crucial to address the possibility of false positives and false negatives. False negative results could delay prompt diagnosis and treatment, putting the patient at potentially fatal risk, while false positive results could cause needless worry and follow-up procedures. Including a follow-up evaluation to gauge the lesions' progression is one practical way to reduce these risks. Furthermore, the accuracy and dependability of computer vision models can be greatly increased by putting additional techniques into practice, such as robustness testing (which involves a variety of lesions, lighting conditions, resolutions, etc.) and human-in-the-loop confirmation by an expert when the model is unsure about the pathology.

## Conclusions

This systematic review outlines the latest advancements in the application of AI to detect OSCC. The findings highlight AI's potential as a valuable tool for early diagnosis, which could enhance patient outcomes. Various AI techniques, including deep learning, computer vision, and machine learning, were utilized in the reviewed studies, with CNNs being the most commonly employed. SVMs and probabilistic neural networks were also implemented in several investigations. Notably, oral photography was the primary method for acquiring image data, and five of the 11 studies reported sensitivity rates exceeding 95%, while four achieved accuracy rates above 95%. Techniques such as texture descriptors, ensemble learning, and multi-task learning have demonstrated promising results, suggesting that AI can improve diagnostic precision even with limited datasets.

Despite AI's potential, its application in oral cancer detection is still in its early stages, requiring further validation through large-scale clinical trials. The most effective approaches appear to either combine CNN with other techniques or avoid CNN entirely, as single attention-based models tend to perform poorly on small datasets. However, advancements in AI training methodologies may overcome these limitations in the future. AI is reshaping cancer diagnostics, enabling the early detection of malignancies through medical imaging modalities such as mammography, CT scans, MRIs, and X-rays. This technological evolution enhances clinical decision-making by identifying subtle abnormalities that human observers may overlook. Ultimately, AI-driven approaches have the potential to improve early diagnosis, facilitate timely interventions, and positively impact patient survival rates in oral cancer management.

## References

[1] Almangush A, Mäkitie AA, Triantafyllou A (2020). Staging and grading of oral squamous cell carcinoma: an update. Oral Oncol.

[2] Kamal F, Ghafary ES, Hamrah MH, Khalid GS, Hamrah MH, Hasam Z, Ghafoory N (2024). Awareness and knowledge of tobacco use and its relation to oral cancer among patients visiting Stomatology Teaching Hospital. Cancer Manag Res.

[3] Khanna D, Shruti T, Tiwari M (2024). Prevalence of oral potentially malignant lesions, tobacco use, and effect of cessation strategies among solid waste management workers in Northern India: a pre-post intervention study. BMC Oral Health.

[4] González-Moles MÁ, Aguilar-Ruiz M, Ramos-García P (2022). Challenges in the early diagnosis of oral cancer, evidence gaps and strategies for improvement: a scoping review of systematic reviews. Cancers (Basel).

[5] Ullah W, Ali Q (2025). Role of artificial intelligence in healthcare settings: a systematic review. J Med Artif Intell.

[6] Umapathy VR, Natarajan PM, Swamikannu B, Jaganathan S, Rajinikanth S, Periyasamy V (2024). Role of artificial intelligence in oral cancer. Adv Public Health.

[7] Veeraraghavan VP, Daniel S, Dasari AK, Aileni KR, Patil C, Patil SR (2024). Harnessing artificial intelligence for predictive modelling in oral oncology: opportunities, challenges, and clinical perspectives. Oral Oncol Rep.

[8] Page MJ, McKenzie JE, Bossuyt PM (2021). The PRISMA 2020 statement: an updated guideline for reporting systematic reviews. BMJ.

[9] Jurczyszyn K, Gedrange T, Kozakiewicz M (2020). Theoretical background to automated diagnosing of oral leukoplakia: a preliminary report. J Healthc Eng.

[10] Xue Z, Yu K, Pearlman PC (2022). Automatic detection of oral lesion measurement ruler toward computer-aided image-based oral cancer screening. Annu Int Conf IEEE Eng Med Biol Soc.

[11] Welikala RA, Remagnino P, Lim JH (2021). Clinically guided trainable soft attention for early detection of oral cancer. Computer Analysis of Images and Patterns.

[12] Welikala RA, Remagnino P, Lim JH (2020). Fine-tuning deep learning architectures for early detection of oral cancer. Mathematical and Computational Oncology.

[13] Tanriver G, Soluk Tekkesin M, Ergen O (2021). Automated detection and classification of oral lesions using deep learning to detect oral potentially malignant disorders. Cancers (Basel).

[14] Ding H, Huang Q, Rodriguez D (2023). Modified locust swarm optimizer for oral cancer diagnosis. Biomed Signal Process Control.

[15] Fu Q, Chen Y, Li Z (2020). A deep learning algorithm for detection of oral cavity squamous cell carcinoma from photographic images: a retrospective study. EClinicalMedicine.

[16] Huang Q, Ding H, Razmjooy N (2023). Optimal deep learning neural network using ISSA for diagnosing the oral cancer. Biomed Signal Process Control.

[17] Shamim MZ, Syed S, Shiblee M, Usman M, Ali SJ, Hussein HS, Farrag M (2022). Automated detection of oral pre-cancerous tongue lesions using deep learning for early diagnosis of oral cavity cancer. Comput J.

[18] Alanazi AA, Khayyat MM, Khayyat MM, Elamin Elnaim BM, Abdel-Khalek S (2022). Intelligent deep learning enabled oral squamous cell carcinoma detection and classification using biomedical images. Comput Intell Neurosci.

[19] Nanditha BR, Geetha Kiran A, Chandrashekar HS, Dinesh MS, Murali S (2021). An ensemble deep neural network approach for oral cancer screening. Int J Online Biomed Eng.

[20] Brocklehurst P, Kujan O, O'Malley LA, Ogden G, Shepherd S, Glenny AM (2013). Screening programmes for the early detection and prevention of oral cancer. Cochrane Database Syst Rev.

[21] Badawy M, Balaha HM, Maklad AS, Almars AM, Elhosseini MA (2023). Revolutionizing oral cancer detection: an approach using Aquila and Gorilla algorithms optimized transfer learning-based CNNs. Biomimetics (Basel).

[22] Gunes H, Piccardi M (2009). Automatic temporal segment detection and affect recognition from face and body display. IEEE Trans Syst Man Cybern B Cybern.

[23] Yang X, Zeng Z, Teo SG, Wang L, Chandrasekhar V, Hoi S (2018). Deep learning for practical image recognition: case study on Kaggle competitions. Proceedings of the 24th ACM SIGKDD International Conference on Knowledge Discovery & Data Mining (KDD '18).

